# The Effects of Low-Power Laser Irradiation on Inflammation and Apoptosis in Submandibular Glands of Diabetes-Induced Rats

**DOI:** 10.1371/journal.pone.0169443

**Published:** 2017-01-18

**Authors:** Cíntia Yuki Fukuoka, Alyne Simões, Toshikazu Uchiyama, Victor Elias Arana-Chavez, Yoshimitsu Abiko, Noboru Kuboyama, Ujjal K. Bhawal

**Affiliations:** 1 Department of Biomaterials and Oral Biology, São Paulo University, School of Dentistry, São Paulo, Brazil; 2 Department of Social Dentistry (Medical Informatics), Nihon University School of Dentistry at Matsudo, Matsudo, Chiba, Japan; 3 Department of Biochemistry and Molecular Biology, Nihon University School of Dentistry at Matsudo, Matsudo, Chiba, Japan; 4 Research Institute of Oral Science, Nihon University School of Dentistry at Matsudo, Matsudo, Chiba, Japan; Universidade de Mogi das Cruzes, BRAZIL

## Abstract

Diabetes can lead to dysfunction of the secretory capacity in salivary glands. Activation of the receptor for advanced glycation end products (RAGE) and its ligands has been suggested to participate in chronic disorders such as diabetes and its complications. In this study, the expression of RAGE, high mobility group box 1 (HMGB1) and advanced glycation end products (AGE), as well as the effects of low-power laser irradiation (LPLI) in salivary glands of diabetic rats were evaluated, and the mechanisms involved were characterized. The expression of RAGE and HMGB1 at the protein and mRNA levels was observed in submandibular glands (SMGs) of streptozotocin-induced diabetic rats. A diode laser was applied at 660 nm, 70 mW, 20 J/cm^2^, 0.56 J/point, with a spot area of 0.028 cm^2^ and its in vivo effects and the pathways involved were evaluated. Immunohistochemistry and western blotting analysis were performed for inflammatory and apoptosis markers. Diabetes up-regulates HMGB1/AGE/RAGE axis gene expression in SMGs that is associated with activation of the nuclear factor kappa B (NF-κB) pathway. Interestingly, LPLI suppresses NF-κB activation induced by inflammation. LPLI also reduces diabetes-induced apoptosis. That effect was accompanied by decreased levels of Bax, and cleaved caspase 3, which were up-regulated in diabetes. Taken together, our data suggest that LPLI reduces diabetes-induced inflammation by reducing the induction of HMGB1, ultimately leading to inhibition of apoptosis in submandibular glands of diabetic rats.

## Introduction

Salivary glands are important exocrine and endocrine organs that contribute to the maintenance of oral and systemic health. Diabetes disrupts homeostasis, resulting in the impairment of salivary glands. Mouth dryness, loss of taste sensation, sialosis, high incidence of caries, tooth loss, periodontal disease and candidiasis have also been reported in diabetic patients [1–4].

High mobility group box 1 (HMGB1) is a chromatin-binding factor that bends DNA and promotes DNA replication and transcription [5, 6]. Extracellular HMGB1 binds with high affinity to the receptor for advanced glycation end products (RAGEs), thereby promoting inflammation [7]. Advanced glycation end products (AGEs) are a heterogeneous group of molecules formed by non-enzymatic reaction glycation or glycoxidation of proteins, lipids and nucleic acids [8]. The accelerated formation of AGEs due to elevated glycemia has repeatedly been reported as a central pathogenic factor in the development of diabetic microvascular complications [9].

Diabetes mellitus is characterized by chronic hyperglycemia. This disturbs homeostasis, leading to the increased formation of AGEs [10] and the accumulation of HMGB1, that binds to RAGE and activates immune cells and the vascular endothelium [11, 12]. RAGE is a unique member of the immunoglobulin superfamily of cell surface pattern-recognition receptor proteins that interacts with a range of ligands, including AGE and HMGB1, which activates the nuclear factor kappa B (NF-κB) signaling cascade [13]. As a result of that activation, the transcription factor NF-κB induces the secretion of pro-inflammatory cytokines, including TNF-α, IL-1β, IFN-γ and IL-6, thereby promoting the recruitment of immune cells that exacerbate and sustain inflammation in a self-perpetuating manner [14, 15]. Therefore, the persistent elevation of RAGE endogenous ligands promotes the sustained activation of NF-κB, leading to chronic inflammation. RAGE modulation is upstream of many important pathological pathways relevant to diabetic complications.

Streptozotocin (STZ) has been used to chemically induce diabetes in experimental models [16]. Although numerous published studies have focused on the role of RAGE and its ligands in diabetes, little is known about the expression of AGE, HMGB1 and RAGE in diabetic submandibular glands (SMGs), as well as the effects of diabetes on apoptosis and proliferation markers in that tissue. Furthermore, in the last decade, low-power laser therapy (LPLI) has been applied clinically for the treatment of hyposalivation [17–20]. Our group previously reported that LPLI increases salivary flow of irradiated rat salivary glands [21] and improves the antioxidant enzyme activities of superoxide dismutase and catalase in SMGs of diabetic rats [22]. We also observed reduced symptoms of hyposalivation after LPLI in patients with head and neck cancer treated with radiotherapy (19), as well as in patients with Sjogren’s syndrome [20]. In vitro, several studies have reported that LPLI modulates various biological processes including cell growth and apoptosis [23–25]. However, the biological molecular mechanism underlying those observed beneficial results remains unclear. Our results demonstrate for the first time that LPLI has potent protective effects against inflammation and apoptosis in diabetes-induced SMGs via its suppression of HMGB1/AGE/RAGE, which in turn inhibits the NF-κB pathway. These findings contribute to a better understanding of the biological functions of LPLI and salivary gland disorders in diabetic conditions.

## Materials and Methods

### Experimental rat model of diabetes

**Fig 1:**
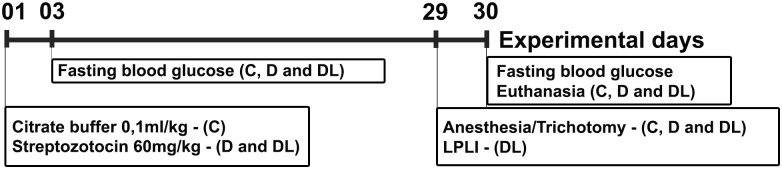
Experimental design. The rats were divided into 3 groups: control (C), diabetes (D) and diabetes + LPLI (DL). On day 1, diabetes was induced in the D and DL groups by injection of STZ. On day 29, the DL group received the LPLI. On day 30, all rats were sacrificed and their SMGs were collected. Each rat’s blood glycemia level was checked on days 3 and 30.

Experimental diabetes-induced rat model was developed as described previously [26]. In brief, female Wistar rats (12-weeks old; n = 30) were purchased from University of Sao Paulo and were housed in isolation cages throughout the experimental period. The rats were allowed access to food and water ad libitum, and they were maintained on a 12 h light/dark cycle (lights on 8:00–20:00) at 22°C. As shown in Fig 1, the rats were randomly divided into 3 groups: control (C)(n = 11), diabetic (D)(n = 9) and diabetic treated with LPLI (DL)(n = 10). Groups D and DL received a 60 mg/kg STZ (Sigma Aldrich, St. Louis, MO, USA) injection intraperitoneally, while group C was administered the same amount of vehicle, citrate buffer pH 4.5. At days 3 and 30, the body weight and blood glucose level of each rat was evaluated. Fasting glycemia was measured by tail blood sampling with a hand-held glucometer (Roche Diagnostics, Mannhein, Germany). Animals with glycemia levels above 250 mg/100 ml were considered diabetics. The rats were sacrificed by cervical dislocation. The experimental procedures of this study were conducted under protocols approved by the Animal Care and Ethics Committee of the Institute of Biomedical Sciences, University of São Paulo, Brazil. All animals were treated in accordance with the principles for animal experimentation established by the Brazilian Committee for Animal Experimentation. For the molecular analysis, the SMG of three animals from each group were used for Western blotting of HMGB1, cleaved-caspase-3 and β-actin for antibody screening. The best sample from each group was chosen and used to perform the Western blotting, qRT-PCR, for the all the other genes, following the procedures described below. In addition, three SMG from different animals, one from each group, were used to perform immunohistochemical and double immunofluorescence analysis. In total, for the molecular analysis data we used two animals from each group. One animal for immunohistochemistry and double immunofluorescence, and another animal for western blotting and qRT-PCR.

### Low power laser irradiation

Twenty-four h before sacrifice, all animals were anesthetized with a Ketamine/Xylazine intraperitoneal injection (4 ml/kg). A trichotomy was performed in the area of irradiation, 1.13 cm^2^ corresponding to the SMGs, and marked with a red circle, resulting in one circle (covering the two SMGs) to be irradiated in each animal. The laser irradiation was applied perpendicular through the skin surface, standardized for all sessions, in a total of 40 points, 70 mW, wavelength of 660 nm, 20 J/cm^2^, 0.56 J/point, 8 sec/point (Photon Lase III, DMC IND. CO., LTD., São Paulo, Brazil), according to Simões et al [27]. The control group was processed under the same conditions, except without the laser irradiation.

### qRT-PCR analysis

Total RNAs were isolated using an RNeasy Mini kit (Qiagen, Hilden, Germany). First-strand cDNAs were synthesized from 1 μg of each total RNA using Super Script VILO Master Mix (Life Technologies, CA, USA). Two μl of each cDNA was subjected to real-time RT-PCR using TaqMan Gene Expression Assays (Applied Biosystems, EUA) for HMGB1 and RAGE, and Pre-Developed TaqMan Assay Reagents (Applied Biosystems, CA, USA) for β-actin as an internal control. Two independent measurements were averaged and relative gene expression levels were calculated as a ratio to β-actin expression of each sample.

### Western blotting

Tissues were removed from RNA Later (Life Technologies) and lysed in RIPA lysis buffer (Santa Cruz Biotechnology, Santa Cruz, CA, USA). Protein concentrations were determined using a BCA Protein Assay Kit (Pierce Biotechnology, Rockford, IL, USA). SDS–PAGE was calibrated with molecular weight markers (Bio-Rad, EUA) for 15 min, and the specimens were rinsed with Tris-buffered saline with 0.1% Tween 20 (TBS-T). Antibodies to HMGB1 (1:800; Abcam, Cambridge, MA, USA), AGE (1:250; Transgenic, Kobe, Japan), RAGE (1:100; GeneTex, Irvine, CA, USA), Bax (1:1000; Abcam, cleaved caspase-3 (Asp175) (1:500; Cell Signaling Technology, Danvers, MA, USA), p53 (1:500; Abcam), phospho-p53 (Ser 15) (1:900; Cell Signaling Technology), phospho NF-κB p65 (1:200; Bioss, Woburn, MA, USA) and β-actin (1:100; Cell Signaling Technology) were used as primary antibodies. Anti-mouse and anti-rabbit secondary antibodies (Cell Signaling Technology) were each used at a dilution of 1:2000. Bound antibodies were visualized by chemiluminescence using the ECL Plus Western Blotting Detection System (Amersham, Uppsala, Sweden), and images were analyzed using a Luminescent Image Analyzer (LAS-3000; Fuji Film Inc., Kashiwa, Japan).

### Immunohistochemistry

Immunohistochemical staining was performed on 4% formaldehyde-fixed, paraffin-embedded rat SMG specimens. Sections were initially immersed in citrate buffer pH 6.0 (Abcam, Inc., Cambridge, MA, EUA) at 97°C for 20 min, and subsequent steps were performed according to the manufacturer's instructions. After 15 min, the specimens were rinsed with phosphate-buffered saline (PBS). Rabbit monoclonal anti-HMGB1 antibody (1:250; Abcam), mouse monoclonal anti-AGE antibody (1:125; Transgenic), rabbit polyclonal anti-IL-1β (1:200; Santa Cruz Biotechnology), rabbit polyclonal anti-TNF-α (IHC World, Woodstock, MD, USA), rabbit polyclonal anti-phospho NF-κB (1:100; Bioss), rabbit polyclonal anti-RAGE (1:20; GeneTex), rabbit monoclonal anti-Bax (1:250; Abcam), rabbit monoclonal anti-Bad (1:100; Abcam) and rabbit polyclonal anti-cleaved caspase-3 (Asp175) (1:200; Cell Signaling) were used as primary antibodies to detect the immunoreactivity in rat tissues. After overnight incubation to block endogenous peroxidase activity with 3% hydrogen peroxide–methanol at 4°C, the specimens were rinsed with PBS and incubated at room temperature for 1 h with the appropriate secondary antibody Histofine Simple Stain Rat MAX-PO (Multi) (Nichirei Biosciences, Tokyo, Japan). After rinsing with PBS, all specimens were color-developed with diaminobenzidine (DAB) solution (DAKO, North America Inc. Carpinteria, CA, USA) and counterstained with hematoxylin.

### Double immunofluorescence for HMGB1 and Bax

Paraffin sections of the SMGs (4 μm thick) were deparaffinized in xylene and rehydrated in a graded alcohol series. Sections were immersed in citrate buffer pH 6.0 (Abcam, Inc., Cambridge, MA, EUA) at 97°C for 20 min, washed in PBS and immersed in peroxidase blocking solution (DAKO) for 10 min. The samples were then washed in PBS and incubated in protein block solution (DAKO) for 1 h. The first antibody Bax (1:150, Abcam) was prepared with Zenon Alexa Fluor 594 (Molecular Probes, Eugene, OR, USA) according to the manufacturer’s instructions, applied to the samples and incubated in a humidified dark chamber for 2 h. The sections were then washed in PBS for 25 min and were subsequently incubated with the secondary antibody to HMGB1 (1:150, Abcam) prepared with Zenon Alexa Fluor 488 for 2 h. DAPI was used for nuclear counterstaining.

### Statistical analysis

Results of blood glucose levels and body weight are expressed as means ± standard deviation. Statistical differences for different groups were evaluated by Student’s two-tailed T-test. A *P*-value of <0.05 is considered statistically significant.

## Results

### Clinical evaluation

The final mean body weight of diabetic rats decreased 5.44% from the initial body weight (Table 1, *P* = 0.004). Thirty days after the induction of diabetes, the mean serum glucose level of diabetic rats was about 2.4 times greater than that of control animals (*P* < 0.05). LPLI significantly reduced blood glucose levels in diabetic rats (*P* = 0.03).

**Table 1:** Metabolism patterns.

Group	Body weight (g)		Blood Glucose (mg/dl)	
	Initial	Final	Initial	Final
**C**	202 ± 18	232 ± 31	125 ± 23	162 ± 38
**D**	206 ± 14	191 ± 22 *	490 ± 111 *	395 ± 181 *
**DL**	206 ± 12	193 ± 22 *	453 ± 121 *	351 ± 167 * #

Body weight and glucose levels of control (C), diabetes (D) and diabetes treated with LPLI (DL) rats, measured 72 h after the diabetes-induction and on the day of sacrifice. Diabetes significantly increased glycemia levels and reduced the body weight of rats. The blood glucose levels of diabetic rats were significantly reduced after the laser irradiation. (*P < 0.05 vs. control group; # *P* < 0.05 vs. diabetes group). Values are shown as means ±SD.

### Effects of LPLI on HMGB1/ AGE/ RAGE axis

**Fig 2:**
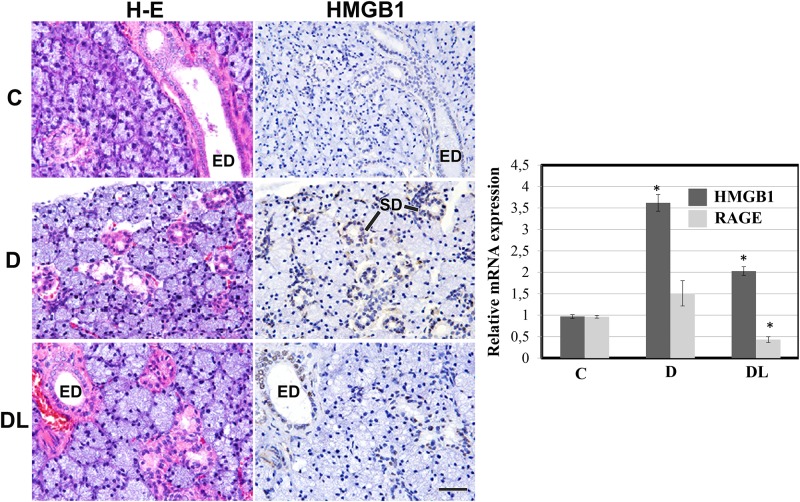
LPLI reduces HMGB1 accumulation in SMGs from diabetic rats. Morphology of SMGs from control, diabetic and diabetic rats treated with LPLI shows typical acinar and ducts cells in all 3 groups. Immunohistochemical analysis of HMGB1 in control, diabetic and diabetic rats treated with LPLI. HMGB1 expression was localized in the nuclei of striated duct cells (arrows), as well as, in the nuclei of endothelial cells in SMGs of diabetic rats. C, Control; D, Diabetes; DL, Diabetes + LPLI; ED, excretory duct; SD, striated duct. Scale bars, 50 μM. Diabetes increases RAGE and HMGB1 mRNA levels analyzed by qRT-PCR, and LPLI reduces those levels to patterns similar to the control group. Two independent measurements were averaged and relative gene expression levels were calculated as a ratio to β-actin expression of each sample.

**Fig 3:**
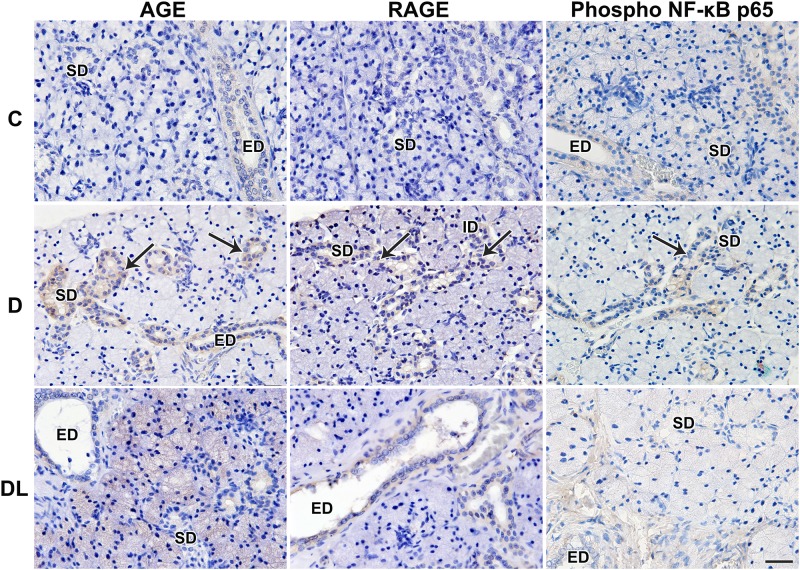
LPLI attenuates inflammation via NF-κB. The expression of AGE, RAGE and phospho NF-κB p65 is observed only in diabetic rats (arrows), in striated and excretory duct cell cytoplasm, with little expression of AGE and RAGE in the DL group and no expression of phospho NF-κB after LPLI. C, Control; D, Diabetes; DL, Diabetes + LPLI; ED, excretory duct; SD, striated duct; ID, intercalated duct. Scale bars, 50 μM.

**Fig 4:**
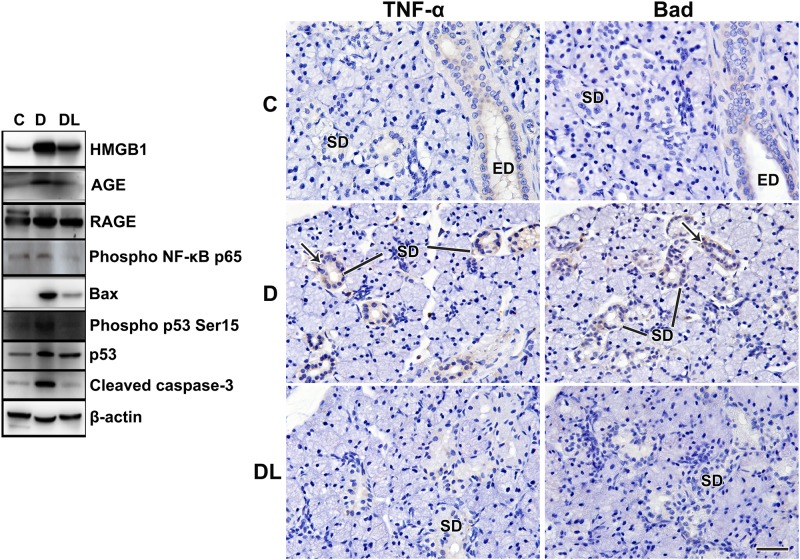
LPLI decreases the expression of cell death markers. Western blotting analysis showed that inflammatory markers (HMGB1, AGE, RAGE and phospho NF-κB p65) and apoptotic markers (Bax, p53, phospho-p53 (Ser15) and cleaved caspase-3) were increased in diabetic rats and were reduced by LPLI. Protein sizes were evaluated by standard protein markers, and their sizes were as follows: HMGB1 (25 kDa), AGE (non-specified), RAGE (46 kDa), phospho NF-κB p65 (65kDa), Bax (21 kDa), phospho-p53 (Ser15) (53 kDa), p53 (53 kDa), cleaved caspase-3 (17–19 kDa) and β-actin (42 kDa). TNF-α and Bad were assessed by immunohistochemistry. There was no expression of TNF-α or Bad in the control group. TNF-α and Bad were both strongly stained in striated duct cell cytoplasm (arrows) in SMGs from diabetic rats, but can barely be seen after LPLI (Fig 4). C, Control; D, Diabetes; DL, Diabetes + LPLI; ED, excretory duct; SD, striated duct. Scale bars, 50 μM.

Typical acinar and duct cells were seen in SMGs from all 3 groups of rats. The mRNA levels of RAGE and HMGB1 increased in diabetic rats (Fig 2). In addition, HMGB1, AGE and RAGE were also observed in SMGs of diabetic rats (Figs 2 and 3). Treatment with LPLI reduced the diabetes-induced accumulation of HMGB1, AGE and RAGE expression (Figs 2 and 3). In order to understand the mechanism of the LPLI-mediated effects, we analyzed RAGE signaling by western blotting and found that LPLI attenuated the diabetes-induced activation of NF-κB (Figs 3 and 4), down-regulating the expression of those inflammatory markers.

### Effects of LPLI on apoptotic markers in SMGs of diabetic rats

**Fig 5:**
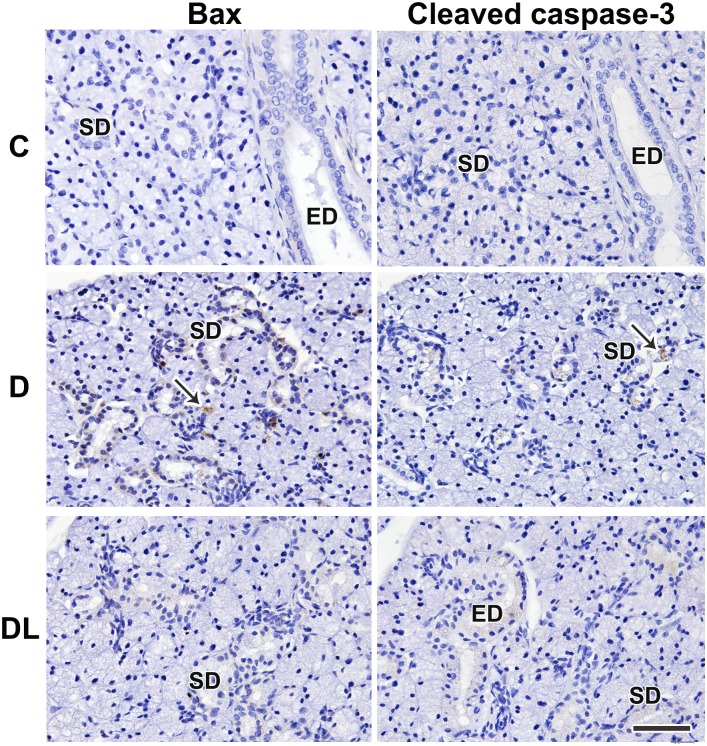
LPLI attenuates apoptosis. Bax and cleaved caspase-3 were assessed by immunohistochemistry. There is no expression of Bax or cleaved caspase-3 in control SMGs. Both markers were localized in SMGs from the diabetic rats, and were strongly stained in striated duct cell cytoplasm (arrows), but after LPLI there was no expression of Bax and only a faint expression of cleaved caspase-3. C, Control; D, Diabetes; DL, Diabetes + LPLI; ED, excretory duct; SD, striated duct. Scale bars, 50 μM.

The expression of apoptosis-related markers, such as TNF-α, Bax, Bad and cleaved caspase-3, was assessed by immunohistochemistry and Western blotting (Fig 4). TNF-α, Bad, Bax and cleaved caspase-3 were localized in diabetic SMGs, and were strongly stained in striated duct cell cytoplasm, without expression in the control group. LPLI-treated diabetic rats had attenuated expression of those apoptosis-related markers (Figs 4 and 5). This was also observed by western blotting analysis for Bax and cleaved caspase-3 (Fig 4). LPLI also reduced total p53 protein levels and its phosphorylation at serine 15 (Fig 4).

**Fig 6:**
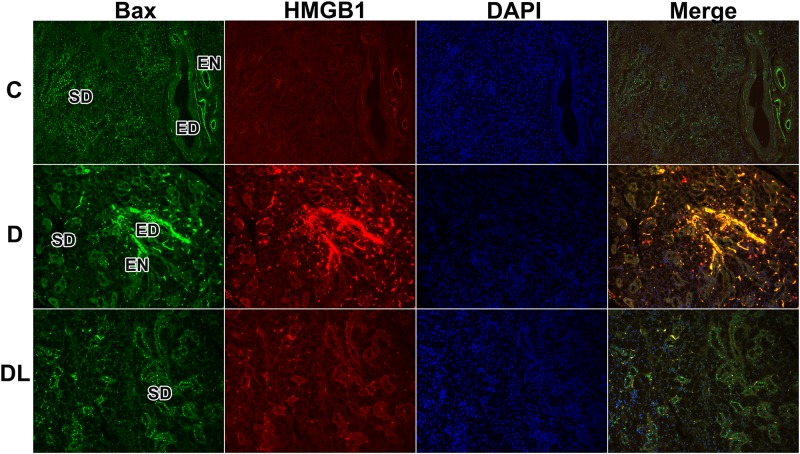
Co-localization of Bax and HMGB1 assessed by double immunofluorescence staining. Double-positive cells were faintly localized in striated and excretory duct cells in the control SMGs, with intense staining in duct cells, as well as in endothelial cells of diabetic rat SMGs. These expression patterns are attenuated by LPLI. C, Control; D, Diabetes; DL, Diabetes + LPLI; ED, excretory duct; SD, striated duct; EN, endothelium. Original magnification 20X.

To further characterize the relationship between HMGB1 and cell death, double immunofluorescence for Bax and HMGB1 was performed. We found the same cellular localization, strong staining in striated and excretory duct cells as well as in endothelial cells of SMGs from diabetic rats (Fig 6). LPLI attenuated the expression of those markers (Fig 6).

## Discussion

The current study is the first to investigate the role of HMGB1/AGE/RAGE in SMGs from diabetic rats and to characterize the underlying mechanism of diabetes complications in that tissue. By employing a diabetic rat model treated with LPLI, we also provide clues for the pathways involved in the effects of LPLI by regulating RAGE ligands/NF-κB molecules, which alleviates diabetic-induced alterations in SMGs.

Diabetes induces inflammation by increasing the expression of RAGE and two endogenous RAGE ligands, HMGB1 and AGE, which activates the downstream NF-κB pathway in SMGs in this rat experimental model. Recent studies have described that hyperglycemia increases RAGE, S100A8 and HMGB1 expression in aortic endothelial cells, both in vitro and in vivo [28]. Yu et al. showed that STZ-induced diabetic rat hearts express increased AGEs accumulation and RAGE expression, raised the levels of TNF-α and IL-1β, and enhanced apoptotic cell death [29]. Alves et al. found a greater expression of AGE, RAGE and NF-κB in lacrimal glands of diabetic rats in comparison with the control [30]. Those studies are consistent with ours and indicate that diabetes up-regulates the AGE/HMGB1/RAGE signaling pathway via the activation of NF-κB, and that those factors are involved in the subsequent inflammatory alterations related to diabetes complications in these organs.

In contrast to the short and self-terminating activation of NF-κB in acute-phase response situations [31, 32], the activation of NF-κB has been suggested to participate in chronic disorders, including diabetic patients with a new onset of diabetes or a long history of diabetes [15, 33, 34]. Recent evidence has demonstrated that the RAGE-mediated sustained activation of NF-κB, which depends on the de novo transcription of NF-κB p65, results in a perpetuated NF-κB-dependent expression [15]. Our study shows the activation of NF-κB in diabetic SMGs, as well as the up-regulation of NF-κB-target genes, TNF-α and RAGE [14]. This animal model supports the hypothesis that RAGE and its ligands are central for the activation of NF-κB in diabetes in vivo.

Interestingly, treatment with LPLI decreases the diabetes-induced transcription of HMGB1 and RAGE, with reductions of AGE, RAGE, HMGB1 and TNF-α protein expression, possibly down-regulating NF-κB in diabetic SMGs, indicating that LPLI has a potent anti-inflammatory effect. This is consistent with a recent report that LPLI inhibits the expression of pro-inflammatory cytokines cyclooxygenase, interleukin-1β and interleukin-6 in human adipose-derived stem cells by down-regulating NF-κB nuclear translocation and its transcriptional activity. Zhang et al. also reported that LPLI decreases NF-κB expression in the synovial membrane in a rat model of rheumatoid arthritis [35].

We also found a significantly decrease in animals blood glucose levels, this is consistent with our previous report, that shows that LPLI can improve the insulin sensitivity in diabetic rats, without changing the levels of HMGB1 or insulin in blood serum [26]. We observed the LPLI effect at 24 h, that provided large amounts of favorable data on inflammation and apoptosis in diabetic SMGs, however it must be considered that these findings may not translate to outcome of long-term studies. Further studies are required to elucidate the novelty of our data in a long-term effect of LPLI.

In our study, diabetes also seems to increase apoptosis in SMG. Hyperglycemia exacerbates glucose oxidation and mitochondrial generation of ROS, which causes strand breaks in nuclear DNA and contributes to accelerated apoptosis [36–38]. The results of our study suggest that diabetes might increase the expression of Bax, Bad and cleaved caspase-3 expression, as well as the phospho-p53 at ser15, an early step in p53 activation [39], all of which are pro-apoptotic proteins. Enhanced apoptotic cell death was also reported in diabetic cardiomyopathy [29, 40] and in diabetic wound healing [41]. AGE can also induce caspase-mediated apoptosis via TNF-α and oxidative stress in bone marrow stem cells [42]. These pro-apoptotic markers are attenuated by LPLI in diabetic tissues; however, this should be studied in more detail.

Our results indicate for the first time that LPLI reduces inflammation and apoptosis in diabetic SMGs. These effects deserve further studies that can deepen our understanding of the mechanism of LPLI, since it can mediate anti-inflammatory responses and bring a promising new therapeutic horizon for salivary gland disorders.
